# Smurf1 Inhibits Mesenchymal Stem Cell Proliferation and Differentiation into Osteoblasts through JunB Degradation

**DOI:** 10.1002/jbmr.28

**Published:** 2010-01-15

**Authors:** Lan Zhao, Jian Huang, Ruolin Guo, Yi Wang, Di Chen, Lianping Xing

**Affiliations:** 1 Department of Pathology and Laboratory Medicine, University of Rochester Medical CenterRochester, NY, USA; 2 Center for Musculoskeletal Research, University of Rochester Medical CenterRochester, NY, USA

**Keywords:** MSC, ubiguitination, proteasome, JunB, osteoblasts

## Abstract

Ubiquitin ligase Smurf1-deficient mice develop an increased-bone-mass phenotype in an age-dependent manner. It was reported that such a bone-mass increase is related to enhanced activities of differentiated osteoblasts. Although osteoblasts are of mesenchymal stem cell (MSC) origin and MSC proliferation and differentiation can have significant impacts on bone formation, it remains largely unknown whether regulation of MSCs plays a role in the bone-mass increase of Smurf1-deficient mice. In this study we found that bone marrow mesenchymal progenitor cells from *Smurf1*^*−/−*^ mice form significantly increased alkaline phosphatase–positive colonies, indicating roles of MSC proliferation and differentiation in bone-mass accrual of *Smurf1*^*−/−*^ mice. Interestingly, *Smurf1*^*−/−*^ cells have an elevated protein level of AP-1 transcription factor JunB. Biochemical experiments demonstrate that Smurf1 interacts with JunB through the PY motif and targets JunB protein for ubiquitination and proteasomal degradation. Indeed, Smurf1-deficient MSCs have higher proliferation rates, consistent with the facts that *cyclin D1* mRNA and protein both are increased in *Smurf1*^*−/−*^ cells and JunB can induce *cyclinD1* promoter. Moreover, JunB overexpression induces osteoblast differentiation, shown by higher expression of osteoblast markers, and JunB knock-down not only decreases osteoblast differentiation but also restores the osteogenic potential to wild-type level in *Smurf1*^*−/−*^ cells. In conclusion, our results suggest that Smurf1 negatively regulates MSC proliferation and differentiation by controlling JunB turnover through an ubiquitin-proteasome pathway. © 2010 American Society for Bone and Mineral Research.

## Introduction

Skeletal homeostasis determines bone mass in adults by achieving balance between bone resorption by osteoclasts and bone formation by osteoblasts.(1) Thus the number and activities of both osteoclasts and osteoblasts must be tightly regulated to ensure proper development and maintenance of the skeletal system. Because osteoblasts are mesenchyme-originated cells, their formation is influenced by the regulation of mesenchymal stem cell (MSC) commitment and differentiation potential. For example, osteoblastic transcription regulator Runx2 is specifically expressed in MSCs prefiguring the skeleton at E10.5.(2,3) Therefore, it is important to study how MSCs commit to osteoblast precursors to obtain clues to curing bone diseases such as osteoporosis.

Smurf1 was identified as a homologous to the E6-AP carboxyl terminus (HECT)-type ubiquitin ligase that was demonstrated to target Smad1,(4) Smad5,(5) Runx2,(6) RhoA,(7) bone morphogenetic protein (BMP) or transforming growth factor β (TGF-β) type I receptors,(8,9) MEKK2,(13) Talin head,(10) and Prickle 1(11) for ubiquitination and degradation. Interaction between Smurf1 and its target protein requires WW domains in the Smurf1 and PY motif of the substrate, a well-known feature of HECT-type ubiquitin ligases.(12) The phyisologic role of Smurf1 in vivo is characterized in *Smurf1*^*−/−*^ mice that display an age-dependent bone mass increase.(13) Intriguingly, the increased bone mass in *Smurf1*^*−/−*^ mice was proposed to be associated with enhanced osteoblast activities, contributed by activation of the JNK signaling cascade rather than an accumulation of BMP signaling factors. Currently available analyses of *Smurf1*^*−/−*^ mice have been focused on differentiated and mature osteoblasts.(13,14) Whether or not Smurf1 affects the function of MSCs or osteoprogenitors remains to be explored.

In this study we demonstrated that bone marrow stromal cells derived from adult *Smurf1*^*−/−*^ mice have increased osteogenic colony formation and a concurrent higher level of JunB protein. Our work demonstrated that JunB is an ubiquitination substrate targeted by Smurf1 as well as a stimulator of MSC proliferation and differentiation into osteoblasts. Therefore, the increased-bone-mass phenotype of *Smurf1*^*−/−*^ mice could be attributed to the absence of JunB ubiquitination machinery and the consequent accumulation of JunB proteins in cells, as evidenced by our siRNA experiments that restored the osteogenic potential of *Smurf1*^*−/−*^ cells to the wild-type level. Collectively, our results suggest that Smurf1 negatively regulates MSC proliferation and differentiation to osteoblasts by controlling JunB protein stability through ubiquitination and the proteasome pathway.

## Materials and Methods

### Animals

All animals used in this study, except those used for isolating calvarial preosteoblasts, were 6 to 10 months old when reduced bone volume occurred in *Smurf1*^*−/−*^ mice, as described previously.(13,14) *Smurf1*^*−/−*^ and wild-type control mice were from a C57/BL6 background. The Institutional Animal Care and Use Committee approved all studies.

### Plasmids and antibodies

*Myc-Smurf1*, *Myc-Smurf1-C710A*, *Flag-Smad6*, and *HA-Ubiquitin* expression vectors were described previously.(15,16) *GST-Smurf1* vector was ordered from Addgene (Cambridge, MA). *pMX-JunB-IRES-EGFP* retroviral vector was obtained from Dr K Matsuo (Tokyo, Japan).(17) We constructed a *Flag-JunB* overexpression vector using *p3XFLAG-CMV-7.1* expression vector (Sigma, St Louis, MO, USA) as the backbone. The tyrosine residue of the PPVY motif of *JunB* was mutated into phenylalanine to generate the *JunB-YF* mutant using a QuikChange Site-Directed Mutagenesis Kit (Stratagene, La Jolla, CA). Monoclonal antibodies specific for Flag, HA, and β-Actin antibodies were purchased from Sigma; anti-JunB, anti-c-Jun, and anti-c-Myc antibodies were from Santa Cruz Biotechnology (Santa Crus, CA, USA); Allophycocyanin (APC)–anti-CD45.2 was bought from eBioscience (San Diego, CA).

### Cell cultures and mesenchymal stem cell isolation

Primary calvarial cells and bone marrow stromal cells were isolated according to our previously described methods.(14,16) Cells were cultured in α minimal essential medium (α-MEM) with 10% to 20% fetal bovine serum (FBS). For isolating MSCs, bone marrow stromal cells were cultured in α-MEM plus 20% FBS, and the second to third passage cells were used. Cells were incubated with anti-CD45 antibody–conjugated microbeads (Miltenyi Biotec, Auburn, CA). The CD45-negative (CD45^−^) population was isolated by negative selection according to the manufacturer's instructions as MSCs. Fluorescence-activated cell sorting (FACS) analyses confirmed that more than 98% of isolated cells were CD45^−^.

### Colony-forming unit (CFU) assays and cell proliferation assays

Bone marrow cells were isolated from the femurs of mice, filtered, seeded as 1 × 10^7^ cells per 15-cm dish in α-MEM plus 15% FBS with β-glycerophosphate. After 25 to 28 days, cells were fixed in 10% formalin and then subjected to alkaline phosphatase (ALP) staining and eosin staining. The numbers of ALP^+^ and total colonies (each containing more than 20 cells) were counted. Cell proliferation assays were performed using CellTiter 96 AQueous One Solution Cell Proliferation Assay (MTS) from Promega (Madison, WI). Triplicates were done for each group.

### GST pull-down, immunoprecipitation, and ubiquitination assays

GST-Smurf1 protein was expressed in Rosetta2 (DE3) and Glutathione S-Transferase (GST) fusion proteins were purified as described previously.(18) In vitro transcription/translation of JunB protein was performed using a TNT Coupled Reticulocyte Lysate System (Promega) with *pRK5-JunB* as the template according to the manufacturer's instruction. Purified GST-Smurf1 proteins were incubated with in vitro–translated JunB protein at 4°C for 3 hours and washed with buffer containing 50 mM of Tris-HCl, pH 7.4, 150 mM of NaCl, 0.5% Nonidet P-40, and 10% glycerol three times. The bound protein were resolved by SDS-PAGE analysis and subjected to Western blotting. For Flag immunoprecipitation (IP), 293T cells were transfected with the indicated plasmids for 48 hours, harvested, and lysed in IP buffer containing 50 mM of Tris-HCl, pH 7.4, 150 mM of NaCl, 0.5% Nonidet P-40, and 10% glycerol. Whole-cell lysates were incubated with 1 µL of anti-Flag antibodies and 20 µL of protein G–agarose (Sigma) for 3 to 4 hours at 4°C. For endogenous IP, C2C12 cells were lysed in IP buffer and incubated with JunB antibodies and 20 µL of protein G–agarose (Sigma) for 3 to 4 hours at 4°C. The immunoprecipitates then were washed with IP buffer, resuspended in sample buffer, and subjected to Western blot analysis. For ubiquitination assays, cells were transfected with HA-ubiquitin, Myc-Smurf1, and either Flag-JunB or Flag-JunB-YF and treated with MG132 (10 µM) for 4 hours before being harvested. Cell lysates were incubated with an anti-Flag antibody and protein G–agarose (Sigma) overnight at 4°C. The immunoprecipitates were subjected to Western blot analysis with an anti-HA antibody.

### BrdU incorporation

*Smurf1*^*−/−*^ and wild-type mice were labeled in vivo with BrdU twice by i.p. injection (a 16-hour interval) and euthanized 2 hours after the second injection. Red blood cells were removed from bone marrow cells of the mice by NH_4_Cl, and the remaining cells were stained with APC–anti-CD45.2 and fluorescein isothiocyanate (FITC)–anti-BrdU for flow analyses using a FITC Brdu Flow Kit (BD Pharmingen, Sparks, MD) according to the manufacturer's instructions. APC–anti-CD45.2 was used for immunofluorescent staining of cell surface marker CD45.

### siRNA

*JunB* siRNA and control siRNA were purchased from Santa Cruz Biotechnology. Calvarial cells were transfected using transfection reagent and medium from Santa Cruz Biotechnology according to the manufacturer's instructions. MSC transfections were performed using DharmaFECT 1 (Thermo Scientific, Pittsburgh, PA) according to the manufacturer's instructions.

### Statistics analysis

Data are presented as means ± SD, and all experiments were repeated at least twice with similar results. Statistical analyses were performed with Stat View Statistical Software (SAS, Cary, NC, USA). Differences between the two groups were compared using unpaired Student's *t* tests, and more than two groups were compared using one-way ANOVA, followed by a Bonferroni/Dunnet test. *p* Values of less than .05 were considered to be statistically significant.

## Results

### Bone marrow mesenchymal stromal cells from *Smurf1*^*−/−*^ mice have increased potential to form osteogenic colonies

**Fig. 1:**
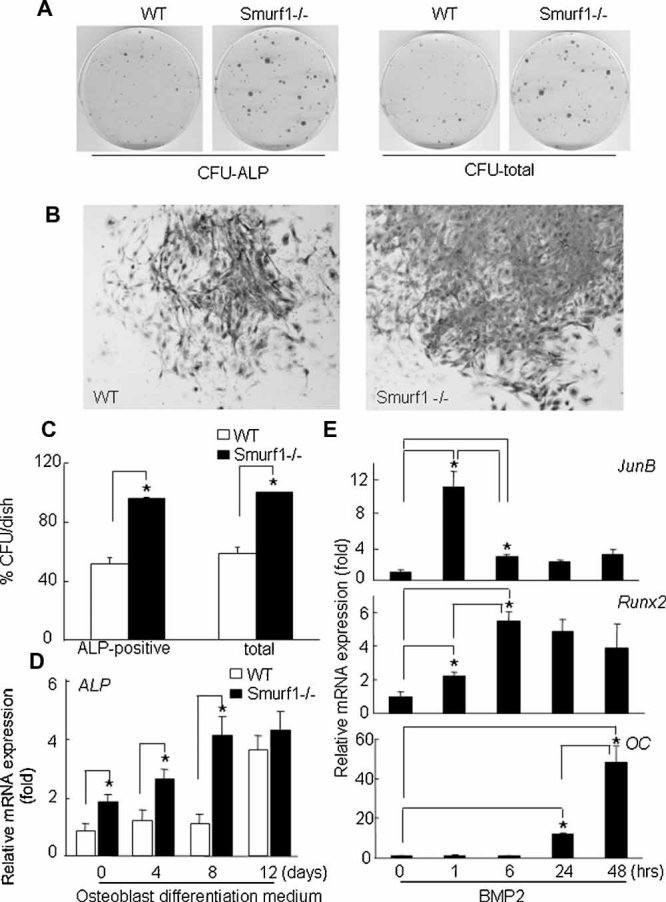
*Smurf1^−/−^* mice have increased bone marrow–derived osteogenic colony formation. (*A*) Representative dishes of ALP^+^ or total colonies formed by wild-type and *Smurf1^−/−^* bone marrow stromal cells. Colony-forming unit (CFU) assays were performed as described in “Materials and Methods.” CFU-ALP, the CFU assays stained with BCIP/NBT, showed ALP^+^ colonies; CFU-total = the CFU assays counterstained with eosin. (*B*) Representative microscopic (×4) images of wild-type or *Smurf1^−/−^* colonies stained with BCIP/NBT and eosin. (*C*) Statistical analyses of CFU assays. The number of colonies (each containing a minimum of 20 cells) was calculated after the total number of *Smurf1^−/−^* colonies was set to 100%. Bars represent mean ± SD of three dishes per genotype. *p < .05 versus wild-type cells, *n* = 3. (*D*) Bone marrow stromal cells from wild-type and *Smurf1^−/−^* mice were cultured in osteoblast differentiation medium, and *ALP* mRNA levels were examined at different time points using quantitative RT-PCR. *p < .05 versus wild-type cells, *n* = 3. (*E*) C2C12 cells were treated with BMP2 (100 ng/mL) with indicated time. *Osteocalcin*, *JunB*, and *Runx2* mRNA levels were examined using real-time qPCR. *p < .05 versus time 0, *n* = 3.

*Smurf1*^*−/−*^ mice develop increased bone mass and osteoblast activities in an age-dependent manner.(13) However, it is not clear whether such phenotypic changes are related to an alteration in MSC proliferation and differentiation into osteoblasts. To assess the proliferation and differentiation potential of MSC osteoblast lineage, we performed a colony-forming assay using bone marrow mesenchymal stromal cells from 6- to 10-month-old *Smurf1*^*−/−*^ and wild-type mice that have developed age-associated bone loss. Specifically, we cultured bone marrow mesenchymal stromal cells with osteogenic medium and measured the number and size of ALP^+^ colonies. The CFU assay results showed that the numbers of both ALP^+^ colonies and total colonies were significantly increased in cells from *Smurf1*^*−/−*^ mice (Fig. 1*A, C*). Generally, *Smurf1*^*−/−*^ cells formed larger colonies than their wild-type counterparts (Fig. 1*A, B*), and the percentage of ALP^+^ cells within one colony was higher (Fig. 1*B*). We also performed real-time quantitative PCR (qPCR) to examine the expression level of *ALP*, an important osteoblast marker gene, and demonstrated that *Smurf1*^*−/−*^ bone marrow stromal cells had higher *ALP* expression levels than wild-type cells with the same duration of osteogenic treatment, suggesting that osteoblast differentiation occurred earlier in *Smurf1*^*−/−*^ cells than in wild-type cells under osteogenic culture conditions (Fig. 1*D*).

Bone marrow stromal cells contain MSCs that give rise to osteoblasts on differentiation, a process that consists of multiple stages and is controlled by important transcription factors including Runx2 and AP-1 family members.(1) Since the *Smurf1*^*−/−*^ phenotype of mesenchymal cell proliferation and differentiation suggests that the regulation may occur at the early stages of osteogenesis, we aimed to search for the transcriptional factors that respond to osteogenic treatment early and intensively. We treated C2C12 myoblast/osteoblast progenitor cells with bone morphogenetic protein 2 (BMP2) and examined the expression levels of *Runx2* and *JunB*, two genes that are known to participate in early BMP responsiveness.(19) The expression levels of *JunB* mRNA peaked (12-fold) at 1 hour after BMP2 treatment and returned to 2- to 3-fold thereafter and stayed at that level for the rest of the time points (Fig. 1*E*). In addition, *Runx2* mRNA started to increase at 1 hour, peaked at 6 hours, and remained at a relatively high level compared with untreated samples. The expression of *osteocalcin*, an important marker gene of mature osteoblasts, also was examined to confirm that the treatment of C2C12 cells resulted in osteoblast differentiation (Fig. 1*E*).

### Smurf1 promotes JunB protein degradation in vivo and in vitro

**Fig. 2:**
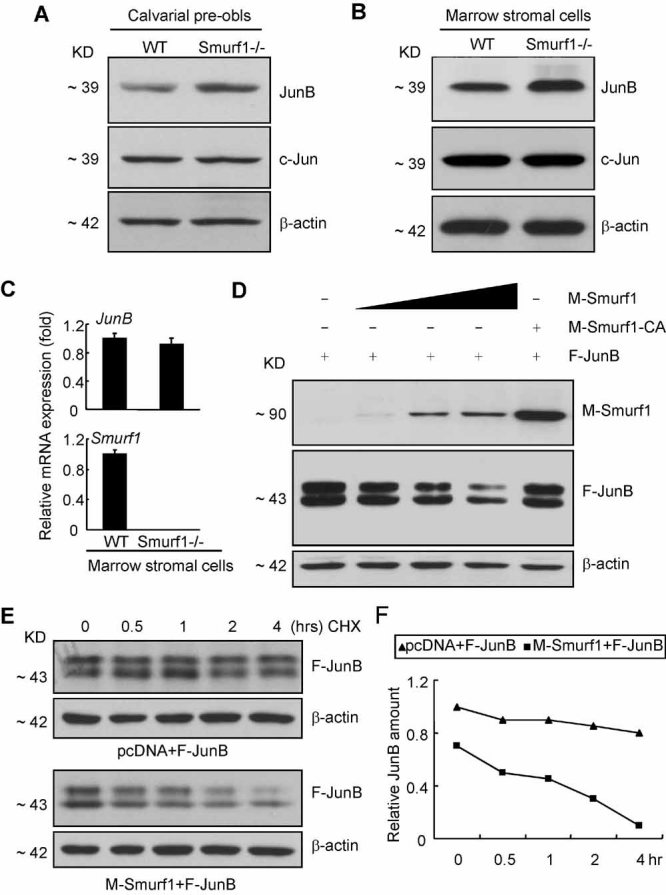
Smurf1 induces JunB degradation in vivo and in vitro. (*A*, *B*) JunB protein level was increased in the primary calvarial (*A*) and bone marrow stromal cells (*B*) of *Smurf1^−/−^* mice compared with that of wild-type mice (*n* = 4). c-Jun protein level also was determined by Western blot analysis. (*C*) Real-time qPCR data showed that *JunB* mRNA level underwent no significant changes in the bone marrow stromal cells of *Smurf1^−/−^* and wild-type mice (*n* = 3). (*D*) Smurf1 expression decreases JunB protein levels in a dose-dependent manner (*n* = 5). HEK293T cells were transfected with *Flag-JunB* and *Myc-Smurf1* plasmids. *Myc-Smurf1* was added in different amounts. *Myc-Smurf1* (C710A) is a mutated Smurf1 in which an active-site cysteine in its catalytic domain is converted into alanine to abolish its activity as an ubiquitin ligase. (*E*, *F*) HEK293T cells transfected with *Myc-Smurf1* and *Flag-JunB* were treated with protein translation inhibitor cycloheximide (80 µg/mL) for indicated time and subjected to Western blot analysis of JunB protein levels to determine the half-life of JunB proteins in the presence or absence of Smurf1 overexpression in HEK293T cells (*n* = 3). Both Western blot (*E*) and its quantification (*F*) are shown.

To determine whether an alteration in JunB expression levels contributes to the osteoblast phenotype of *Smurf1*^*−/−*^ mice, we performed Western blot analyses using cell lysates from *Smurf1*^*−/−*^ bone marrow stromal cells or calvarial preosteoblasts. The results showed that the protein levels of JunB were increased in *Smurf1*^*−/−*^ cells (Fig. 2*A, B*), whereas the expression levels of *JunB* mRNA from the marrow stromal cells were not changed (Fig. 2*C*), suggesting that JunB protein levels may be regulated by Smurf1. Another AP-1 transcription factor, c-Jun, underwent no changes in both mRNA and protein levels (data not shown; Fig. 2*A, B*).

Smurf1 is a HECT-type E3 ubiquitin ligase that promotes the ubiquitination of its target protein through recognizing a PY motif of the target protein. We found that both human and mouse JunB proteins contain the PPXY sequence (Supplemental Fig. 1). To determine whether forced expression of Smurf1 induces JunB protein degradation, we cotransfected *Myc-tagged Smurf1* (*M-Smurf1*) and *Flag-tagged JunB* (*F-JunB*) expression vectors into 293T cells and performed Western blot analysis to examine JunB protein levels with or without Smurf1 overexpression. Significantly, Smurf1 expression reduced JunB protein levels greatly, whereas the mutant Smurf1 (Smurf1-C710A), of which ubiquitin ligase function was rendered catalytically inactive by mutation of cystine 710 to alanine, could not decrease JunB protein levels (Fig. 2*D*). Furthermore, we found that the Smurf1-induced JunB protein decrease occurs in a dose-dependent manner (Fig. 2*D*). Since AP-1 factors can be regulated by mitogen-activated protein kinase (MAPK), we treated cells with JNK, Erk, or p38 MAPK inhibitors and found that the inhibition of MAPKs have no effect on Smurf1-induced JunB degradation in vitro (Supplemental Fig. 2; and data not shown). We also performed a cycloheximide decay assay to study whether the turnover rate of JunB protein was changed by Smurf1 overexpression. As Fig. 2*E, F* shows, the half-life of JunB protein was decreased dramatically in Smurf1-overexpressing cells; meanwhile, the JunB protein was much more stable when Smurf1 was not overexpressed.

### Smurf1 interacts with JunB through PY motif and induces its ubiquitination and degradation

**Fig. 3:**
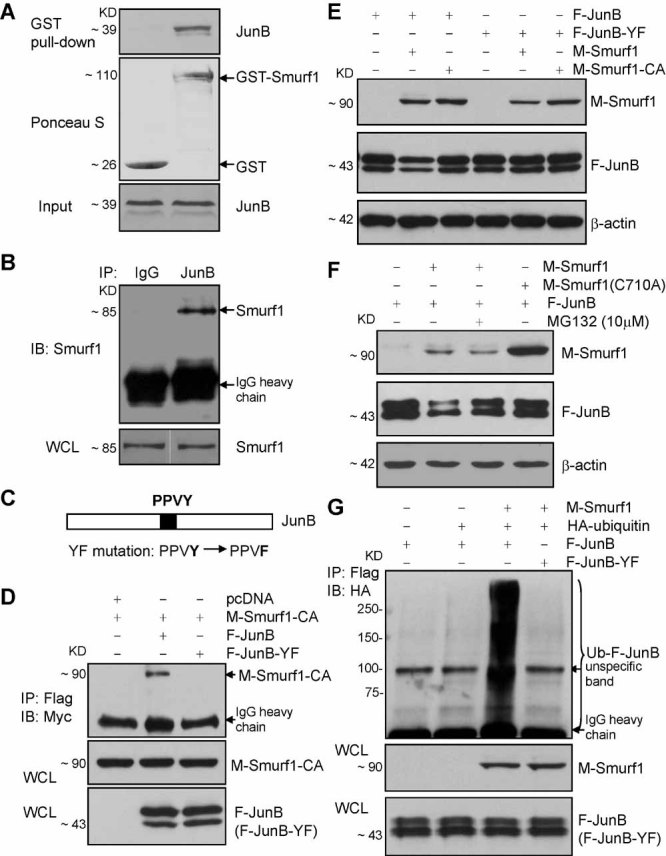
Smurf1 interacts with JunB through a PY motif and induces its ubiquitination and degradation. (*A*) GST-Smurf1 directly interacts with JunB protein. In vitro–translated JunB protein was copurified with GST-Smurf1 in a GST pull-down assay (*n* = 3). (*B*) JunB-Smurf1 interaction in mesenchymal C2C12 cells. Smurf1 was identified in the immunoprecipitates by anti-JunB antibodies (*n* = 3). (*C*) A diagram showing a YF mutation in the PY motif of *JunB* that was generated to examine if the PY motif is required by Smurf1-induced JunB degradation. (*D*) Wild-type JunB protein interacts with Smurf1, whereas JunB (YF) mutant cannot (*n* = 3). HEK293T cells were transfected with *Myc-Smurf1-C710A* and *Flag-JunB* or *Flag-JunB-YF*. IP assays were carried out using anti-Flag antibodies, and immunoprecipitates were subjected to Western blot using anti-Myc antibodies; whole-cell lysates also were examined to ensure that both JunB and Smurf1 were expressed. (*E*) Smurf1 overexpression could not decrease the protein level of JunB-YF (*n* = 4). HEK293T cells were transfected with plasmids as indicated. (*F*) Proteasome inhibitor MG132 rescued JunB protein from Smurf1-induced degradation (*n* = 3). HEK293T cells were transfected with plasmids as shown. Cells transfected with *Myc-Smurf1* and *Flag-JunB* were treated with MG132 (10 µM) for 4 hours before being harvested and subjected to Western blot analysis. (*G*) JunB ubiquitination assay (*n* = 3). 293T cells transfected with *HA-ubiquitin*, *Myc-Smurf1*, and *Flag-JunB* or *Flag-JunB-YF* were treated with MG132 for 4 hours before harvesting, and polyubiquitinated JunB was immunoprecipitated by anti-Flag and immunoblotted with anti-HA.

Smurf1, like other HECT-type E3 ubiquitin ligases, consists of N-terminal C2 domain, WW domains, and a catalytic C-terminal HECT domain. The WW domains of E3 ligases bind the PPXY motif of a target protein to mediate subsequent ubiquitination and degradation. To examine whether Smurf1 binds JunB proteins in a direct manner, we performed the GST pull-down assay using purified GST-Smurf1 protein and in vitro translated JunB protein. The pull-down results showed that JunB protein can be copurified with GST-Smurf1 (Fig. 3*A*), indicating a direct interaction between Smurf1 and JunB. We also confirmed that Smurf1-JunB interaction occurs in C2C12 myoblast/osteoblast progenitor cells by IP of both Smurf1 and JunB proteins using anti-JunB antibodies (Fig. 3*B*). To examine whether Smurf1 interacts with JunB through the WW-PPXY interface, we generated a JunB mutant in which the PPVY motif in JunB protein was mutated to PPVF using a PCR-based site-directed mutagenesis method, resulting in a mutant named JunB-YF (Fig. 3*C*). Then we used IP assays to examine the bindings of Smurf1 to wild-type JunB and the JunB-YF mutant. Since Smurf1-C710A has greater stability than wild-type Smurf1 and the C710A mutation does not impair the protein-protein interaction domains, Smurf1-C710A was used in the IP assays. Wild-type JunB, but not JunB-YF, can be immunopurified with Smurf1, indicating that Smurf1 binds JunB directly through the PPVY motif of JunB protein (Fig. 3*D*). Compared with wild-type JunB, whose levels were decreased by overexpression of Smurf1, the JunB-YF protein levels were unchanged in the same experiment (Fig. 3*E*), suggesting that a direct interaction between Smurf1 and JunB is required for Smurf1-induced JunB degradation.

Since Smurf1 specifically interacts with JunB and induces its degradation, we reasoned that JunB can be polyubiquitinated when Smurf1 is present. To demonstrate that Smurf1-mediated JunB degradation is through the ubiquitin-proteasome system, we examined whether proteasome inhibitor MG132 blocks JunB degradation. As expected, after treatment with MG132 for 4 hours, the degradation of JunB was prevented (Fig. 3*F*), indicating that Smurf1-induced JunB degradation is proteasome-dependent. To demonstrate that Smurf1 promotes ubiquitination of JunB protein, we performed a ubiquitination assay. 293T cells were cotransfected with *Myc-Smurf1*, *HA-Ubiquitin*, *Flag-JunB*, or *Flag-JunB-YF* mutant and treated with MG132. IP, using an anti-Flag antibody, followed by immunoblotting with an anti-HA antibody, showed that the wilt-type JunB, but not the JunB-YF mutant, resulted in polyubiquitination (Fig. 3*G*).

### Smurf1 has a negative effect on the proliferation of bone marrow mesenchymal stem cells

**Fig. 4:**
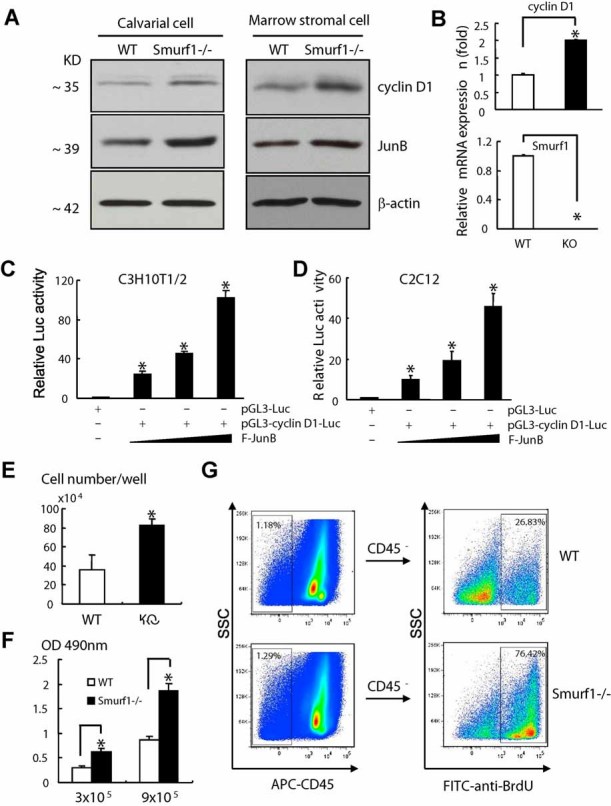
Smurf1 negatively regulates MSC proliferation. (*A*) Western blot results showed that the cyclin D1 protein level was increased in the primary calvarial cells or in the marrow stromal cells of *Smurf1^−/−^* mice compared with wild-type mice (*n* = 3). (*B*) Real-time qPCR data showed that the *cyclin D1* mRNA level was increased in marrow stromal cells of *Smurf1^−/−^* mice. *p < .05 versus wild-type cells, *n* = 3. (*C*) C3H10T1/2 mesenchymal stem cells were transfected with *cyclin D1* reporter and different doses of *JunB* overexpression plasmids. Luciferase activities showed that *cyclin* *D1* promoter was induced by JunB expression in a dose-dependent manner. *p < .05 versus *pGL3-Luc*, *n* = 3. (*D*) C2C12 myoblast/osteoblast progenitor cells were transfected with *cyclin D1* reporter and different doses of *JunB* overexpression plasmids. Luciferase activities showed that *cyclin D1* promoter was induced by JunB expression in a dose-dependent manner. *p < .05 versus *pGL3-Luc*, *n* = 3. (*E*) The numbers of *Smurf1^−/−^* bone marrow stromal cells were higher than those of wild-type cells after 7 days of culture in 12-well plates. *p < .05 versus wild-type cells, *n* = 4. (*F*) Cell proliferation rates were increased in *Smurf1^−/−^* BMSCs. Thus 3 × 10^5^ or 9 × 10^5^ BMSCs per well were seeded into 96-well plate, and cell proliferation was examined using a colorimetric assay. *p < .05 versus wild-type cells, *n* = 4. (*G*) *Smurf1^−/−^* CD45^−^ MSCs have a higher proliferation rate than wild-type cells. Bone marrow cells were stained with APC-CD45 and FITC–anti-BrdU after in vivo labeling of BrdU by i.p. injection. CD45^−^ and BrdU^+^ populations of wild-type and *Smurf1^−/−^* cells were analyzed (*n* = 3).

It has been reported that JunB plays an important role in osteoblasts. Calvarial preosteoblasts and bone marrow stromal cells from JunB-deficient mice have decreased proliferation and differentiation.(20,21) Since our Western results demonstrated that both calvarial preosteoblasts and bone marrow stromal cells of *Smurf1*^*−/−*^ mice have accumulated JunB proteins, it would be interesting to examine whether these cells have increased proliferation and elevated cell-cycle protein such as cyclin D1, a positive regulator of cell proliferation. Thus we performed Western blot to examine cyclin D1 protein levels in calvarial preosteoblasts and bone marrow stromal cells and found that these cells had higher cyclin D1 protein levels than wild-type cells (Fig. 4*A*). Next, we examined the mRNA expression of *cyclin D1* and found that *cyclin D1* transcription was induced at a higher level in *Smurf1*^*−/−*^ marrow stromal cells that also have elevated JunB protein (Fig. 4*B*). In fact, it was reported that *cyclin D1* promoter contains binding sites of AP-1 factors, through which JunB regulates *cyclin D1* expression in fibroblasts(22,23) and in mesenchymal C2C12 cells (Supplemental Fig. 3). To determine whether JunB activates *cyclin D1* promoter, we transfected the *cyclin D1-Luc* reporter construct into the mesenchymal stem cell line C3H10T1/2 cells and studied whether JunB introduction enhances the *cyclin D1* promoter reporter activities. As Fig. 4*C* shows, *cyclin D1* promoter responded to JunB expression in a dose-dependent manner, suggesting that JunB promotes *cyclin D1* expression at a transcriptional level in MSCs. Similar results also were obtained when C2C12 cells were used (Fig. 4*D*), indicating that increased osteogenic colonies in the *Smurf1*^*−/−*^ mesenchymal cells shown in Fig. 1*A*–*C* may be due to increased proliferation. To test this, we decided to examine the proliferation in MSCs in vitro and in vivo. We counted the cell numbers of bone marrow stromal cells cultured in vitro and found that the number of *Smurf1*^*−/−*^ cells was significantly higher than that of wild-type cells (Fig. 4*E*), although the bone marrow cells were plated in the same number. Cell proliferation assays also confirmed an increased proliferation rate of *Smurf1*^*−/−*^ bone marrow stromal cells (Fig. 4*F*). We labeled *Smurf1*^*−/−*^ bone marrow cells with BrdU by i.p. injection and stained them with a combination of APC–anti-CD45 and FITC–anti-BrdU and then performed FACS analyses to assess the percentage of BrdU^+^ cells within the CD45^−^ cell population. Murine bone marrow MSCs, characterized as lineage^−^/CD45^−^/CD105^+^, have been used for osteoblast differentiation.(24) We demonstrate that CD45^−^ cells isolated from adult mouse marrow are hematopoietic lineage^−^, and half of them are CD105^+^ after culturing in vitro (Supplemental Fig. 4). Furthermore, CD45^−^ cells can give rise to osteoblasts and adipocytes, whereas CD45^+^ cells cannot (data not shown). Based on this fact, we can gate out the CD45^+^ hematopoietic lineage cells from bone marrow cells and analyze the CD45^−^ MSC population specifically. Flow cytometric results demonstrated that BrdU^+^ cells comprised 76.42% of CD45^−^ cells in *Smurf1*^*−/−*^ MSCs and were significantly more enriched than those (26.83%) in wild-type MSCs (Fig. 4*G*), suggesting that Smurf1 deficiency positively regulated MSC proliferation.

### JunB degradation induced by Smurf1 also negatively regulates MSC differentiation into osteoblasts

**Fig. 5:**
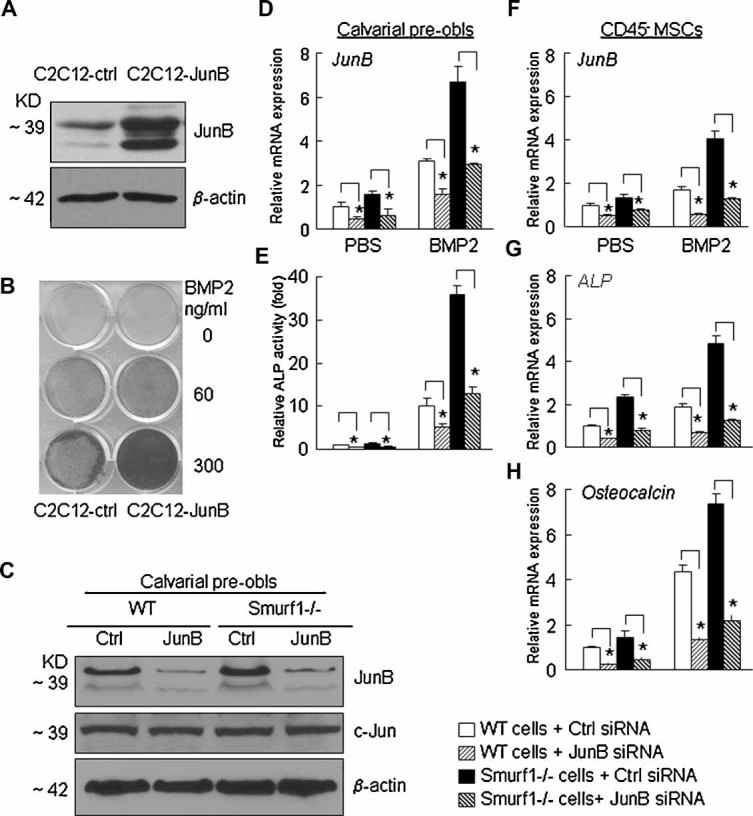
Smurf1-induced JunB degradation has a negative impact on osteoblast differentiation of MSCs. (*A*) Western blot showed that the JunB protein level was increased in C2C12 cells infected with *JunB-*overexpressing retrovirus (*n* = 3). (*B*) Enhanced ALP activity by JunB overexpression in C2C12 myoblast/osteoblast progenitor cells shown by ALP staining of control or JunB-overexpressing cells on BMP2 treatment at the doses indicated (*n* = 3). (*C*, *D*) *JunB* knockdown by siRNA was confirmed by Western blotting (*C*) or RT qPCR (*D*). *p < .05 versus wild-type cells + control siRNA, *n* = 3. Calvarial cells of wild-type and *Smurf1^−/−^* mice were isolated and transfected with *JunB* siRNA or control siRNA. Cells were cultured in osteoblast differentiation medium with or without BMP2. (*E*) ALP activity of the calvarial cells transfected with siRNAs was measured to evaluate whether *JunB* knockdown restored increased osteogenic differentiation in *Smurf1^−/−^* cells. *p < .05 versus wild-type cells + control siRNA, *n* = 3. (*F–H*) CD45^−^ MSCs were isolated from in vitro–cultured bone marrow stromal cells of wild-type and *Smurf1^−/−^* mice, transfected with *JunB* siRNA or control siRNA and then cultured in osteoblast differentiation medium with or without BMP2. *JunB* (*F*), *ALP* (*G*) and *osteocalcin* (*H*) mRNA levels were measured by real-time qPCR. *p < .05 versus wild-type cells + control siRNA, *n* = 3.

An increase in osteogenic potential and JunB protein levels in *Smurf1*^*−/−*^ bone marrow stromal cells suggests a positive correlation between osteoblast differentiation and JunB expression levels. To confirm this, we overexpressed JunB protein in C2C12 cells, a myoblast/osteoblast precursor cell line, by retrovirally infecting cells with *JunB*-expressing retrovirus. As the JunB protein level increased in C2C12 cells (Fig. 5*A*), ALP activity also was strikingly induced in these cells (Fig. 5*B*), suggesting that enhanced JunB protein level increased osteoblast differentiation potential of the progenitor cells. This result is consistent with the studies performed using *Smurf1*^*−/−*^ cells. Next, to further confirm that JunB is responsible for the increased osteoblast differentiation potential of *Smurf1*^*−/−*^ cells, we preformed *JunB* RNAi experiments to investigate whether *JunB* knockdown can restore increased osteoblast differentiation of *Smurf1*^*−/−*^ cells with or without BMP2 stimulation. We isolated calvarial preosteoblasts and purified the CD45^−^ MSC population from bone marrow stromal cells using CD45 antibody–conjugated magnetic beads and transfected these cells with *JunB* siRNA. Our RNAi experiments achieved considerable downregulation of *JunB* expression: Most samples had over 50% decrease of *JunB* mRNA or protein compared with those transfected with control siRNA (Fig. 5*C, D, F*). It should be noted that *JunB* mRNA expression in wild-type and *Smurf1*^*−/−*^ cells (Fig. 5*D, F*) were not similar, as we had observed in Fig. 2*C*. This discrepancy could be caused by a longer culture of cells to reach a confluence of greater than 90% before siRNA transfection and to minimize the deleterious effects of siRNA transfection. Such a long-term culture could result in MSC differentiation, and *Smurf1*^*−/−*^ cells had greater potential to differentiate into osteoblasts; thus *JunB* mRNA expression was induced in a greater degree in *Smurf1*^*−/−*^ cells, as Fig. 1*E* suggested. Next, we examined ALP activity or *ALP* mRNA expression, as well as *osteocalcin* mRNA expression by RT-PCR to study if osteoblast differentiation was affected in those cells with decreased JunB protein levels. We found that *JunB* siRNA significantly decreased *ALP* mRNA expression or protein activity by 50% to 60% under basal and BMP2-stimulated conditions in both calvarial preosteoblasts and CD45^−^ MSCs of wild-type mice (Fig. 5*E, G*). Importantly, *JunB* downregulation by siRNA restored *ALP* expression or activity in both preosteoblasts and CD45^−^ MSC of *Smurf1*^*−/−*^ mice to a level similar with that of wild-type mice (Fig. 5*E, G*), suggesting that Smurf1 deficiency–enhanced osteoblast differentiation of MSCs is JunB-dependent. Examination of the *osteocalcin* mRNA level in CD45^−^ MSCs (Fig. 5*H*) further confirmed that JunB plays a positive role in osteoblast differentiation, which may significantly contribute to Smurf1 deficiency–promoted bone formation.

## Discussion

Using bone marrow stromal cells and purified bone marrow MSCs derived from adult *Smurf1*^*−/−*^ mice, we demonstrated that Smurf1-deficient cells have increased CFU-ALP colony formation and osteoblast marker gene expression. For the underlying mechanism, we demonstrated that Smurf1 regulates the protein stability of *JunB*, an immediate-early gene induced by BMP2. Smurf1 binds directly to *JunB* and promotes its ubiquitination and proteasomal degradation. Smurf1 deficiency affects both differentiation and proliferation of bone marrow MSCs, which is rescued by *JunB* knockdown. Thus the inhibitory effect of Smurf1 on osteoblast function occurs at the stem cell level through the regulation of JunB protein degradation.

*JunB* is a member of the AP-1 family and is an immediate-early gene induced by BMP2 in C2C12 myoblast/osteoblast progenitor cells.(19) Loss of *JunB* affects vasculogenic and angiogenic processes in the developing placenta,(25) causing embryonic lethality, which can be rescued by intercrossing *junB*^+/−^ with Ubi-*junB* transgenic mice or crossing *junB-*floxed mice with mice carring the *MORE-Cre* knock-in allele. Both these JunB-deficient mice are osteopenic. Osteoblasts and precursors derived from bone marrow stromal cells or calvarial cells have reduced proliferation, marker-gene expression, and mineralized bone nodule formation,(20,21) indicating that JunB plays a critical, positive role in osteoblast differentiation, proliferation, and function. In this study, our data demonstrate that JunB is negatively regulated by ubiquitin ligase Smurf1 in MSC proliferation and differentiation into osteoblasts, confirming that JunB functions as an important transcriptional factor in the early stages of bone formation.

Cell proliferation is controlled by many factors, including cyclins. Cyclin D1 is a target of several bone anabolic agents in preosteoblasts.(26,27) For instance, parathyroid hormone–related protein (PTHrP) upregulates cyclin D1 transcription. Finding of JunB stimulates cyclin D1 promoter activity by directly binding the AP-1 response element of the *cyclin D1* promoter in MSCs, indicating that JunB contributes to the increase in cell proliferation through *cyclin D1*. However, we cannot exclude other mechanisms because JunB also regulates the expression of other cell cyclin mediators.(28) Nevertheless, these findings reveal a novel function of ubiquitin ligase Smurf1 in MSC proliferation and differentiation.

Smurf1 is known to control bone mass in aged mice. Overexpression of Smurf1 decreases the protein levels of Smad1 and -5, Runx2, and the BMP receptor, which are involved in osteoblast differentiation. However, calvarial preosteoblasts from *Smurf1*^*−/−*^ mice have normal BMP signaling response, arguing against these BMP signal proteins as the endogenous targets for Smurf1 in these cells.(13) We have demonstrated that Smurf1-induced degradation of BMP signal proteins plays a more important role in inflammatory osteoblast inhibition than in normal bone remodeling.(14) *JunB* is an early response gene for BMP and regulates both cell proliferation and differentiation, whereas Smad1 and -5 and Runx2 mainly affect osteoblast differentiation. Thus, although Smurf1 also ubiquitinates proteins other than JunB, Smurf1-induced changes in JunB protein levels in MSCs and osteoblast precursors likely contribute to increased CFU colony-forming capacity of *Smurf1*^*−/−*^ cells.

JunB ubiquitination and proteasome degradation have been reported in activated T cells by E3 ligase Itch.(29–31) Smurf1-mediated JunB ubiquitination and degradation represent another regulatory mechanism controlling JunB function in cells. Although Smurf1 and Itch both promote JunB degradation, they clearly have different effects on bone cells. *Smurf1*^*−/−*^ mice have an age-related increase in bone mass and osteoblast function.(13,14) In contrast, while *itch*^*−/−*^ mice have decreased bone volume (our preliminary observation), bone quality or osteoblast function in *itch*^*−/−*^ mice has not been investigated. Furthermore, the Itch E3 ligase activity is regulated by phosphorylation. After T cell receptor engagement, Itch undergoes JNK1-mediated phosphorylation that causes configuration changes that greatly enhance its enzymatic activity.(29,30) We have examined the effect of various kinase inhibitors, including Erk, JNK, p38, and AKT, on Smurf1-mediated JunB degradation and found that they have no influence (Supplemental Fig. 2). Interestingly, Smurf1 is restricted to induce the degradation of JunB but not c-Jun proteins in MCSs, whereas Itch was demonstrated to ubiquitinate both c-Jun and JunB proteins in T cells.(29) These results suggest that a specific regulation of JunB ubiquitination and degradation induced by HECT-type ubiquitin ligases occurs in the MSCs to ensure that JunB functions properly in bone-forming process.

Together our results represent a novel facet of bone homeostasis that correlates AP-1 factor and HECT-type ubiquitin ligase regulating MSC proliferation and differentiation, giving clues for developing new therapeutic strategies to prevent age-dependent bone loss.
