# Supplementary material for: Smurf1 Inhibits Mesenchymal Stem Cell Proliferation and Differentiation into Osteoblasts through JunB Degradation
Source: J Bone Miner Res. 2010 Jan 15;25(6):1246–56. doi: 10.1002/jbmr.28 (PMC3153132; doi:10.1002/jbmr.28)
Supplement: Supplementary file 1 [file jbmr0025-1246-SD1.ppt]

## Slide 1
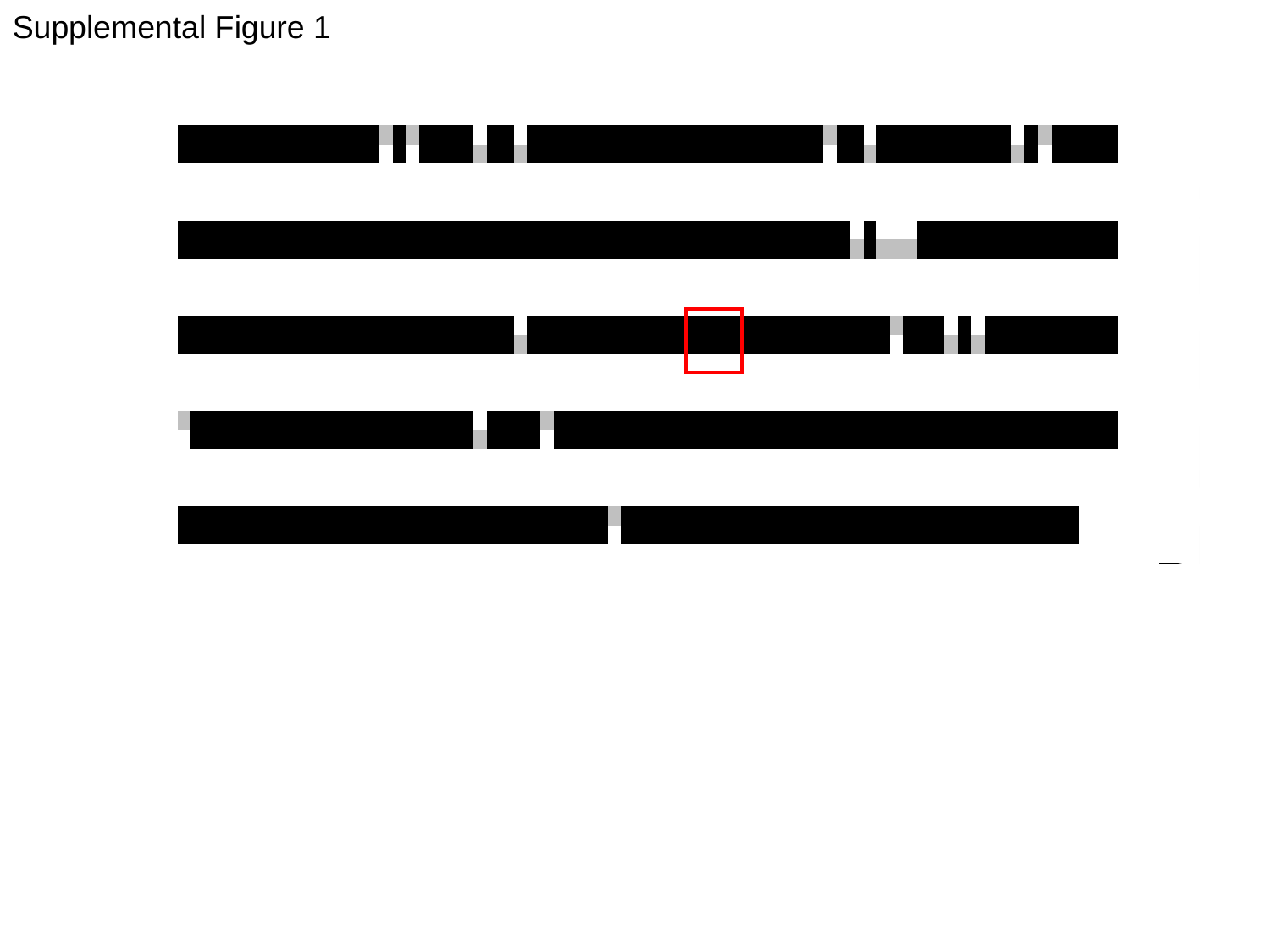

Supplemental Figure 1

## Slide 2
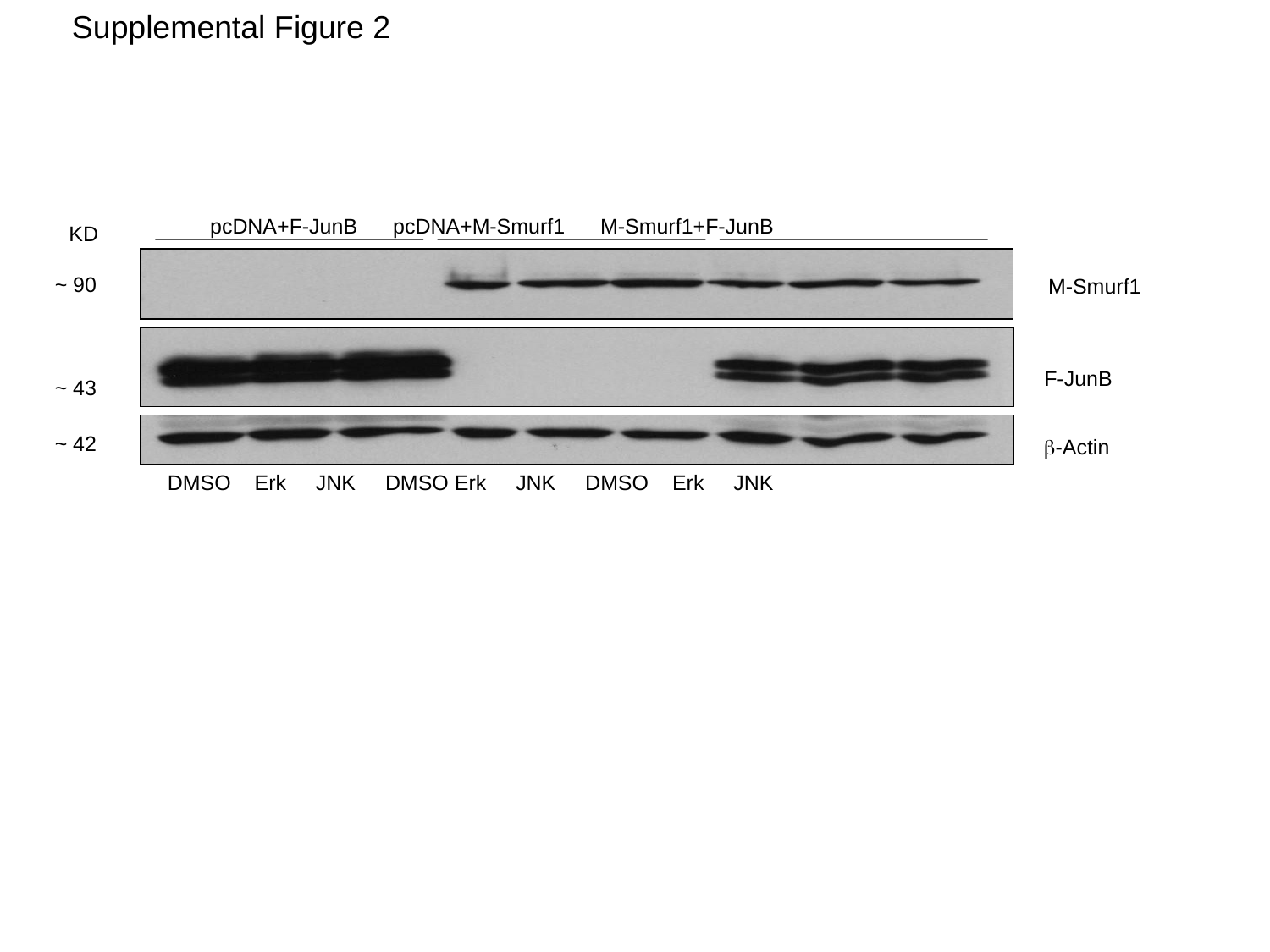

Supplemental Figure 2
pcDNA+F-JunB pcDNA+M-Smurf1 M-Smurf1+F-JunB
KD
~ 90
M-Smurf1
F-JunB
~ 43
~ 42
β-Actin
DMSO Erk JNK DMSO Erk JNK DMSO Erk JNK

## Slide 3
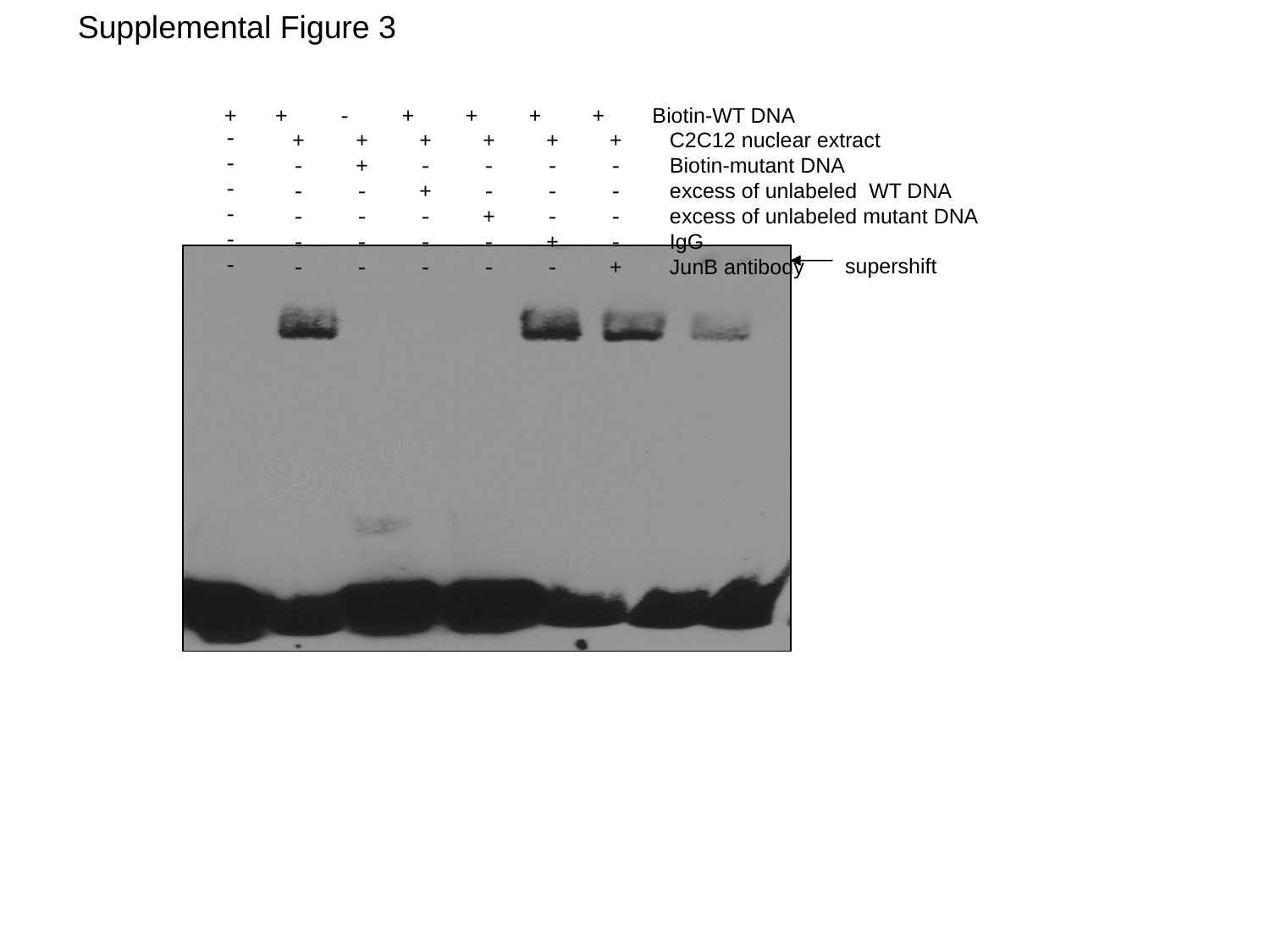

Supplemental Figure 3
+ + - + + + + Biotin-WT DNA
 + + + + + + C2C12 nuclear extract
 - + - - - - Biotin-mutant DNA
 - - + - - - excess of unlabeled WT DNA
 - - - + - - excess of unlabeled mutant DNA
 - - - - + - IgG
 - - - - - + JunB antibody
supershift

## Slide 4
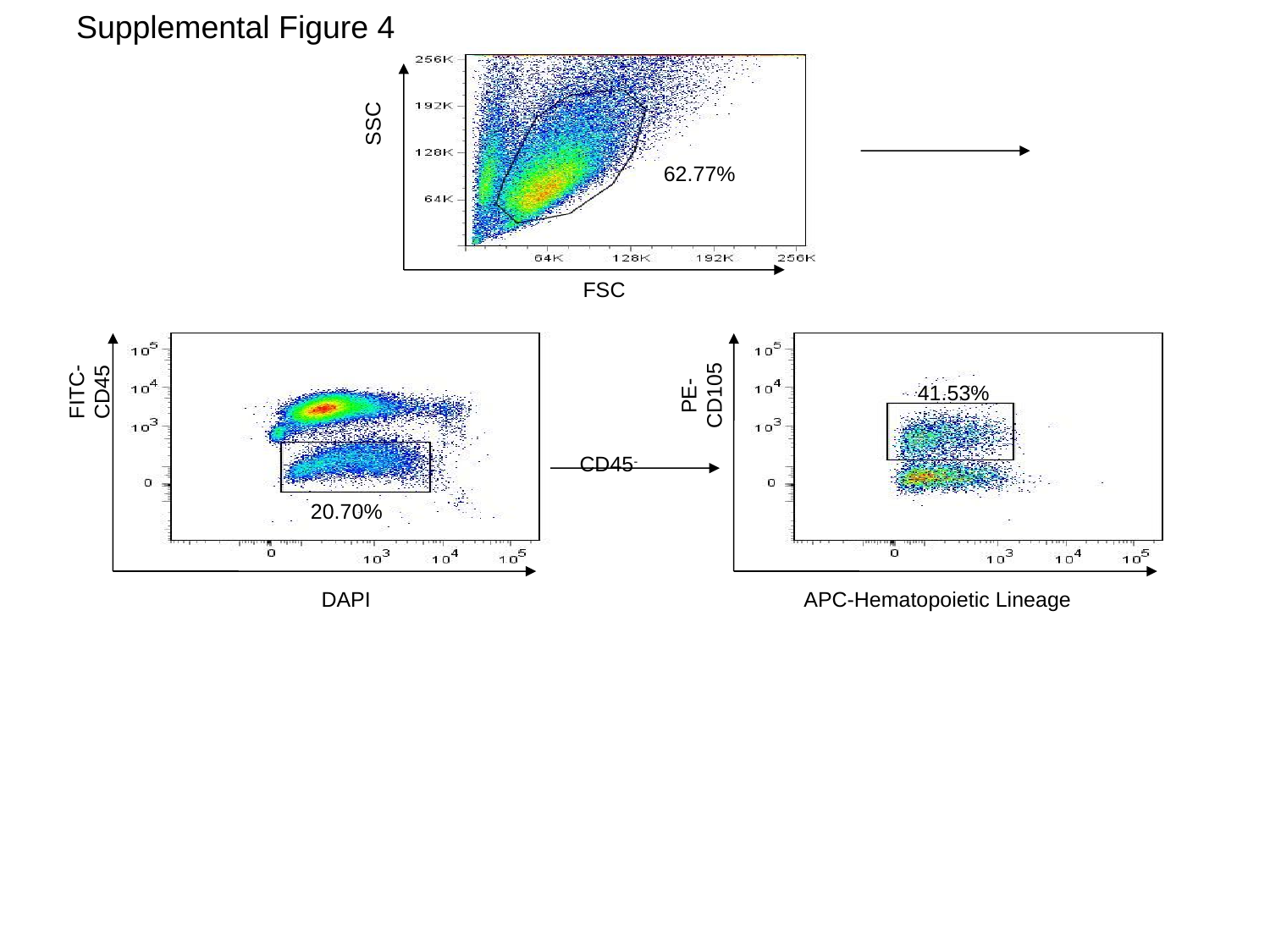

Supplemental Figure 4
SSC
62.77%
FSC
FITC-CD45
PE-CD105
41.53%
CD45-
20.70%
DAPI
APC-Hematopoietic Lineage
